# Enabling community input to improve equity in and access to translational research: The Community Coalition for Equity in Research

**DOI:** 10.1017/cts.2022.396

**Published:** 2022-04-29

**Authors:** Karen M. Emmons, Michael Curry, Rebekka M. Lee, Albert Pless, Shoba Ramanadhan, Carolina Trujillo

**Affiliations:** 1 Department of Social and Behavioral Science, Harvard TH Chan School of Public Health, Boston, MA, USA; 2 The Massachusetts League of Community Health Centers, Boston, MA, USA; 3 Town of Andover, Andover, MA, USA; 4 Volunteer Engagement, Eastern Bank, Boston, MA, USA

**Keywords:** Health equity, community engagement, research participation

## Abstract

**Background:**

The COVID vaccine trials illustrated the critical need for the development of mechanisms to serve as a bridge between least advantaged communities and researchers. Such mechanisms would increase the number of studies that are designed with community needs and interests in mind, in ways that will close gaps rather than widen them. This paper reports on the creation of the Community Coalition for Equity in Research, a community-driven resource designed to build community capacity to provide researchers with credible and actionable input on study design and implementation and increase researchers’ understanding of factors that influence community support of research.

**Methods and Results:**

We provide a description of the Coalition’s structure and process and an evaluation of its first year of operation. Researchers rated their experience very positively and reported that the Coalition’s review will improve their research. Coalition members reported high levels of satisfaction with their participation and the processes set up for them to engage with researchers. Members also largely agreed that their participation has value for their community, and that it has increased their interest in research and the likelihood that they would recommend research participation to others.

**Conclusions:**

The Coalition represents a model for increasing two-way engagement between researchers and the larger community. We are optimistic that the Coalition will continue to develop and grow into a vibrant entity that will bring value to both investigators and our local communities and will increase the consideration of equity as a foundational principle in all translational research.

## Introduction

The COVID-19 pandemic put a public face on two realities that health equity researchers have struggled with for years. First, there are structural factors that pose significant harms to the health and well-being of low-income and historically disadvantaged communities. Every aspect of the pandemic exposed the role of structural determinants of health, including employment-related factors that increased exposure to COVID-19 and reduced the ability to quarantine and shelter in place (e.g., lack of paid sick leave), transportation factors that increased risk while commuting to work without the necessary personal protection equipment and reduced access to many testing programs, as well as housing instability that confronted many furloughed and laid off individuals in low-income communities who were already living at the margins [1,2]. The loss of health and life as a result of the pandemic has been disproportionately born by those whose daily lives are impacted by these structural determinants of poor health, reflecting the distribution of health burden prior to the pandemic [3–5].

Second, although researchers indicate a desire to engage low-income and historically disadvantaged communities in research, they do so infrequently, despite the rich potential and resources that these communities can offer. The COVID vaccine trials faced significant issues with accrual of diverse participants, most notably those from Black, Native, and Latinx backgrounds [6–12], at least in part because the trials were designed without community involvement. The trials were designed and implemented building on long-standing relationships between industry, government, and academia. Historically disadvantaged communities, who have had the highest risk of infection and death due to COVID, were peripheral actors in the pursuit of COVID-19 vaccines [13]. The involvement of the communities bearing the greatest burden of disease began as short-term community “outreach” once the trials had been launched. As several vaccine candidates entered Phase III trials, anticipated acceptability decreased significantly from 54% in May 2020 to 32% in September 2020 among Blacks and from 74% to 56%, respectively, among Latinx individuals [14]. Deeply rooted mistrust resulting from an extensive history of abusive medical experimentation, unequal treatment, and ongoing structural racism, in combination with limited engagement, impedes participation in clinical trials by racially and ethnically diverse participants.

Although the issue of diversity in clinical trials representation has received significant attention in the context of the COVID-19 pandemic, the issue is not new. Almost 30 years ago the NIH Revitalization Act of 1993 mandated appropriate inclusion of minority group members in NIH-funded research, and in particular that there be sufficient representation to allow for valid analysis of different population groups. There has been a significant literature produced in the last three decades demonstrating the limited impact of this legislation [15,16]. Twenty years after the Act was passed, only 2% of 10,000 cancer trials funded by the National Cancer Institute had sufficient minority participation to enable valid analyses by racial or ethnic groups [16]. Of 782 papers of randomized clinical trials published in top tier US medical journals, only 13% reported outcomes by race or ethnicity [17]. A wide array of programs have been recommended to improve diversity in trial recruitment, such as patient education, improved access to interpreters (oral) and/or translators (written), use of plain language consent and informational materials, use of social media, and incentives for participation [18]. The Community Engagement Studio model has been particularly successful in facilitating input from stakeholders on the design, implementation, and dissemination of research [19]. These meetings focus on providing tailored project-specific input from unique panel patients and/or community members aligned with the research topic. Of note, many recommended strategies are focused on the individual level, addressing patient-level factors only. Recently, institutional barriers have also been noted as playing a role in limiting diverse enrollment in clinical trials. In particular, there has been a call for the development of strategies to ensure commitment to diverse enrollment that permeates all institutional structures and roles, rather than resting with a single entity, such as an outreach office [20]. It has also been noted that there is a need for research staff to be diverse, and for systematic training to be provided for clinical research personnel regarding recruitment of diverse participants [21]. These are all important activities that would likely improve trial accrual, if systematically and sustainably implemented.

However, missing from many of these discussions is the creation of an ongoing, bidirectional relationship between researchers and the community that centers equity considerations to inform study development in ways that would potentially increase access to, relevance of, and interest in specific research studies by members of historically disadvantaged communities. Building equity into research involves much more than successful recruitment of a diverse study sample. A recent qualitative study focused on increasing African-American representation in dementia research noted that participants prioritized a two-way flow of information between researchers and participants that is consistent and deeply interwoven [22]. Such a two-way engagement might have helped address many of the key factors that challenged the COVID vaccine trials. For example, trust is challenged by speed, and the tremendous pressure to conduct COVID research quickly led to concerns about whether appropriate protections were in place. When researchers have established relationships with trusted organizations and community leaders, they can be quickly engaged to help identify issues that may erode trust and/or reduce interest in participation. However, trying to create these relationships in the context of trial deployment means that fundamental design issues that impede participation cannot be addressed, and that deep and lasting levels of engagement are not created.

We have argued that the research community has not built the infrastructure needed for trials to be fully responsive to need of diverse communities [13]. Translational researchers often do not invest the time to develop skills and relationships with community partners that would support effective research design for inclusion of historically disadvantaged populations, to understand both the concerns and the rich potential of the community, or to understand the way in which study design features may reinforce structural racism and limit community power. Further, the translational research community has not invested at sufficient levels in building community capacity to more actively engage in research, which would allow for community expertise to be combined with foundational research knowledge in support of providing actionable feedback. Mechanisms are sorely needed to address these gaps and to serve as a bridge between least advantaged communities and researchers. With such mechanisms in place, it would be more likely that research projects are designed with community needs and interests in mind, in ways that will close gaps rather than widen them. We propose that an effort to re-imagine the ways in which we engage communities in research is long overdue. This paper reports on the creation of the Community Coalition for Equity in Research, a community-driven resource designed to build community capacity in ways that provide researchers with credible and actionable input on study design and implementation and increase researchers’ understanding of factors that influence community support of research. The primary goal is to create 2-way engagement and learning opportunities for both researchers and community members, with a focus on improving research design and methods in ways that will maximize both participation and equitable health outcomes.

## Methods

### Program Description

**Fig. 1.**
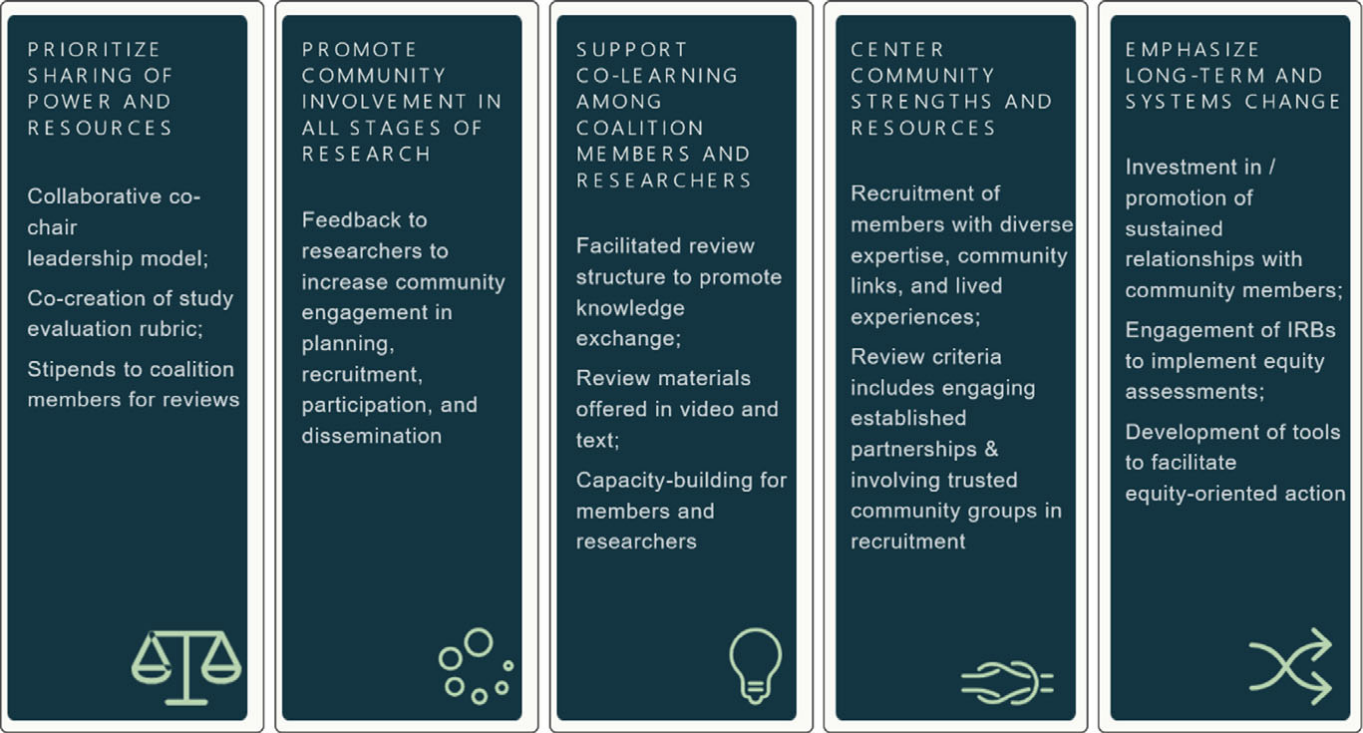
Community-engaged research values aligned with core activities of the Community Coalition for Equity in Research.

The Community Coalition for Equity in Research is a 14-member group of community members who provide feedback on research to promote equity and use of community-engaged research principles. The Coalition is based in the Community Engagement Program at Harvard Catalyst, Harvard’s Clinical and Translational Science Center (CTSA), with shared leadership by an academic researcher (KE) and a community leader and civil rights activist (MC). As planned, two Coalition members were identified to serve as co-chairs (AS, CT), so that there is a balance of leadership between the organizers and Coalition members. The Co-Chairs serve a 1-year term with the possibility for renewal and opportunity for other members to rotate into leadership over time. Two doctoral level members of our Community Engagement Program (RL and SR) and a program coordinator support the activities of the Coalition. The leadership team meets monthly, the week after each Coalition meeting, to debrief on the last meeting and plan for the next session. The leadership team is also responsible for developing initial ideas for new Coalition capacities, which are then discussed, refined, and vetted by the full Coalition. A summary of the principles underlying the Coalition’s structure and processes is provided in Fig. 1.

### Recruitment of Coalition Members

Recruitment for the first cohort of the Coalition was conducted in November 2020 through a self-nomination application process advertised via personal and professional networks, social media, and listservs. Recruitment focused on networks of community members and leaders among communities experiencing health inequities in Massachusetts. The leadership team has been conducting community-engaged work across the state for many years, supporting the ability to tap a diverse set of connections. Prospective members were asked to submit a resume or work/volunteer history, a brief description of the assets they would bring to the Coalition, as well as the ways in which participation would be meaningful to their personal or professional development. Members were selected to maximize diversity across a range of characteristics, including race, ethnicity, gender, sexual orientation, geographic location (targeting regional representation across Massachusetts), and professional experience. Twenty-one people applied to be members of the Coalition cohort and 14 were selected. Those selected had strong ties with diverse community constituencies, were able to articulate their motivations for participating, and as a group represented significant diversity. Members range in age from 25 to 66, with work experience within the health sector (e.g., at health agencies and community health centers) and from outside the health sector (e.g., communications, faith-based organizations, and community development organizations). Most members identify as Black or Latinx and several bring lived experiences as immigrants, members of the LGBTQ community, and residents of historically disadvantaged communities. Biographies of our first cohort are available on our program website: https://catalyst.harvard.edu/community-engagement/community-coalition/leadership-and-membership/.

### Capacity Building and Co-Creation of Coalition Processes and Resources

To ensure that Coalition members were fully prepared to engage in review of research and to provide credible and actionable input, we dedicated the first 3 months of the program (January–March 2021) to activities focused on building research skills and knowledge among community leaders, cultivating a deeper understanding of priority community concerns and ways to improve engagement among researchers, and developing shared group norms and values. Such activities included:

- Brief presentations on and discussion of the clinical trials process and the state of diversity and inclusion in such studies;
- Asynchronous review of the PCORI Research Fundamentals module on “Designing a Research Study” [23];
- Brainstorming session about how the Coalition can have an impact on research;
- Discussion of short-term (e.g., increase transparency, accountability, and initiation of equity-promoting processes) and long-term goals (e.g., research culture shift that centers community) for the Coalition.

In addition to this up-front capacity building for community members, we have designed the Coalition to ensure ongoing joint capacity building for all stakeholders. For instance, prior to review of two genetics-related studies, we provided brief readings and a presentation on the basics of genetics research. We have also compiled a glossary to provide access to the language and culture of research and community-oriented term and acronyms, which is continuously updated to foster communication between researchers and community members (Supplementary Materials). Some Coalition members have shared additional materials for further expansion of their knowledge and that of the group.

One key aspect of the Coalition is the co-creation of our health equity review rubric. After discussion of our impacts and goals, we worked as a group to collaboratively develop a set of equity-focused review criteria – this included a crosswalk of health equity best practices from the literature with the priorities identified by community members in the coalition [24–26]. These 24 criteria (Supplementary Materials) were discussed and refined by the group and ultimately organized into four categories: (1) study planning; (2) recruitment and consent; (3) participation; and (4) dissemination.

During the first year, we identified several areas in which more efforts are needed to promote equity that are common across research projects, such as simplifying recruitment materials and including participants of more diverse language backgrounds. Therefore, we have begun the process of co-creating support materials with Coalition members that can be shared with researchers prior to review and disseminated to a broader researcher audience.

### Researcher Engagement

We identify researchers who may benefit from presenting to the Coalition through our Community Engagement Program consultation service, Harvard Catalyst monthly newsletters, word of mouth via academic networks and Coalition members, advertising directly to faculty and department administrators, and engaging with our institutional IRBs and sponsored program offices. Researchers across rank, disciplines (e.g. medicine, public health), and various research topics and methods (e.g., COVID-19 vaccine trials, genetics research, cardiovascular behavior change interventions) have been engaged. The incentive for researchers to participate is that they may get feedback that will help them reach their diversity recruitment goals, and their findings may be more generalizable if their participants are more representative of the population affected. Ideally, the Coalition will review studies relatively early in their development process, when there is an opportunity for feedback to be integrated into study planning and budgeting. Researchers are asked to prepare the following in advance of presenting to the Coalition: (1) a brief 10-minute video in lay language to provide a broad introduction to the project and the investigator’s motivation for the work proposed; the intention is to complement written materials in order to allow for multiple learning styles and to give the Coalition exposure to the investigator; guidance is provided to the investigator about video preparation for this audience, and sample videos are provided; and (2) a 3-page document to provide detail on study procedures; investigators are asked to respond to key study design questions, as noted in Table 1.

**Table 1.** Components of the written study description provided to the coalition

**What is the primary goal of this study, and why are you conducting it?** **In what ways do you seek to address issues of health equity through these study goals?** **Where will the study be conducted?** **Who are the “participants” in this study and why?** **What are the study outcomes?** **Study planning, including:** (1) study design; (2) any stakeholder relationships that have informed the study design and how that input has been considered; (3) the expertise and diversity of the research team; (4) ways in which study activities consider the social and emotional needs of participants; and (5) ways in which accountability to community partners will be ensured. **Recruitment, including**: (1) recruitment and consent process; (2) involvement of trusted patient advocacy /community groups that serve diverse populations included; (3) incentives; (4) process to develop written materials and languages included. **Participation, including:** (1) the participation burden, including participation costs and (2) study exclusion criteria. **Dissemination, including**: (1) how return of results will be handled; (2) plans for disseminating study findings; (3) the role of and resources for community partners in dissemination activities.

When recruitment and survey materials are developed, researchers are asked to provide them for consideration. Researchers are also asked to prepare specific questions that they would like to ask the Coalition members which may address specific needs as well as providing a place to do initial “field testing” of new strategies that are being considered. Investigators provide materials 2 weeks prior to their meeting with the Coalition, and Catalyst staff provide feedback to investigators to ensure appropriate language, length, and tone. Final materials are provided to the Coalition a week in advance of the meeting, with a request that the study review rubric be completed no later than the day before the meeting. This allows the facilitator the opportunity to review members’ ratings of the study in advance and be prepared for the discussion.

### Review Process

**Fig. 2.**
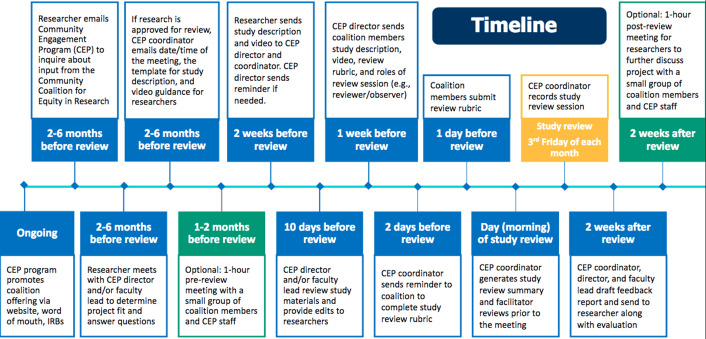
Study review timeline for the Community Coalition for Equity in Research.

Coalition members dedicate 2–4 hours per month to the review of research studies (e.g., for 1 hour meetings, we assume 1 hour of preparation). These hours are compensated at $25/hour and paid as honoraria every other month. For each study, Coalition members independently review the materials provided by the researchers and complete a health equity scoring rubric via a Qualtrics survey form. The first half of the session is a closed discussion among the Coalition members to review the project and prioritize feedback in three areas: (1) feedback on strengths of the project related to health equity; (2) clarifying questions for the researcher(s); and (3) opportunities to improve the project’s health equity focus. In the second half of the meeting, the researcher(s) join for a facilitated conversation and the feedback is discussed, as well as any additional questions the researcher may have regarding further addressing health equity. Coalition members alternate roles as observer or reviewer across sessions. The Coalition reviewers provide feedback directly to the researcher, while observers reflect on the process of the conversation and provide feedback through the chat. After each meeting, staff draft written recommendations for the research team, combining issues raised in the rubric, in the discussion with researchers, in the chat from the meeting, and in follow-up email reflections from Coalition members. The Study Review Timeline is provided in Fig. 2.

The review process was developed to be iterative and flexible in nature. Coalition members provided formal feedback for improvement of the process at the 6-month mark of the first year and informal feedback is built into the end of each meeting. Examples of ways the Coalition review processes have evolved over its first year include:

- Addition of optional pre-review meetings with investigators seeking support prior to review (e.g., advice on preliminary data to be submitted with a grant);
- Addition of optional post-review meetings with investigators seeking additional input (e.g., follow-up on researcher’s specific questions that fall outside of the review rubric);
- Expanded strategies for providing feedback to meet the needs and strengths of Coalition members (e.g., sending reflections by email after review, providing written feedback using the chat feature).

**Fig. 3.**
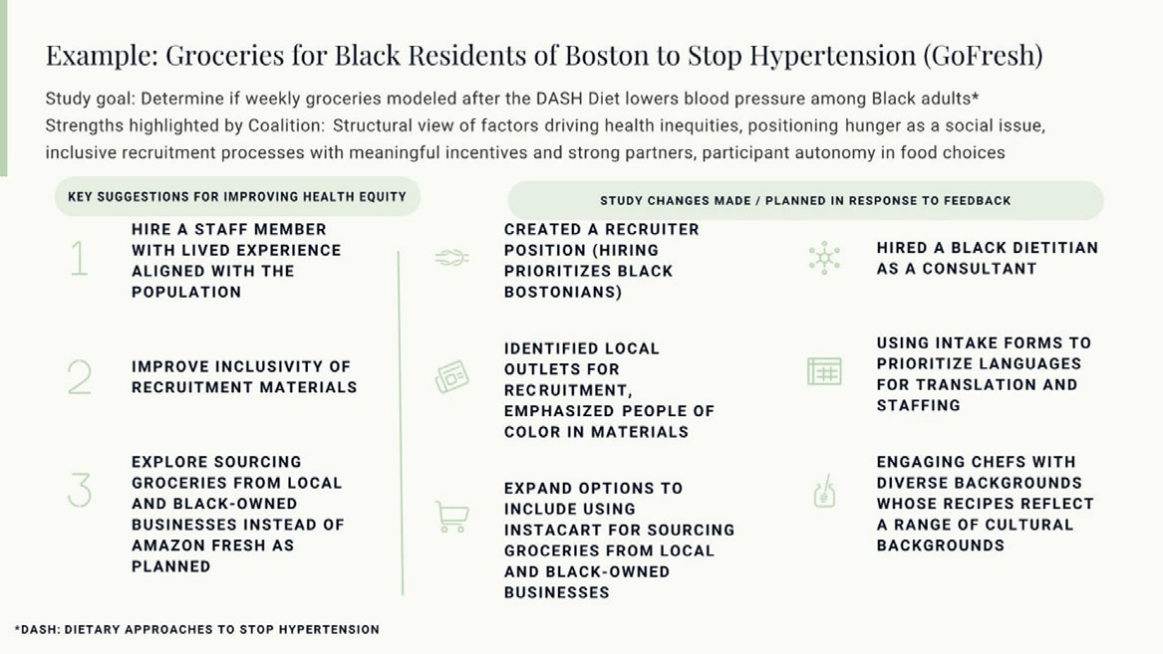
A case example of recommendations made by the coalition, and the changes made to the study procedures.

A case example of the type of feedback provided to researchers is provided in Fig. 3. It illustrates the practical and feasible nature of the recommendations, along with the responsive nature of the changes made to the study procedures.

### Evaluation

#### Measures

We have taken a multipronged approach to evaluating the Coalition. First, review rubrics submitted by Coalition members for each research project capture individual perceptions of the degree to which each of the health equity criteria is considered (e.g., fully, somewhat, not) in each study, as well as qualitative feedback on major health equity strength and top priority changes for addressing health equity. After investigators attend review meetings and receive written feedback, they complete a survey capturing their satisfaction with the Coalition process (e.g., overall assessment, rating of scheduling, facilitation), as well as feedback on the health equity considerations gained in alignment with the rubric criteria. Finally, at the end of the first year, Coalition members completed an on-line survey capturing their satisfaction with the Coalition process, knowledge and skills gained, as well as qualitative feedback on the experience in the first year. This evaluation approach was reviewed by the Office of Human Research Administration and determined to be Not Human Subjects Research.

## Results

### Researcher Evaluation

In our first year, five researchers have presented six research projects to the Coalition for review. All researchers rated their experience as excellent or very good and reported that the coalition included relevant stakeholder experts, that the study review will improve their research, and that coalition member feedback was provided in a respectful and appropriate manner. Areas identified for improvement included allowing more time for feedback and providing the researcher with more information about what to expect before the study review. The most common health equity considerations that researchers reported they planned to change after review were: (1) building and leveraging relationships with community partners during study planning; (2) developing welcoming and inclusive study recruitment activities and language; (3) involving trusted patient advocacy and community groups that serve diverse populations in recruitment; (4) accessibility of recruitment materials; (5) providing opportunities for participation in ways that are tailored to low resource communities of color, leverage trusted partners, and consider the social and emotional needs of participants; and (6) ensuring timely return of results that incorporates needs, preferences, and values of stakeholders.

### Coalition Member Evaluation

The anonymous on-line evaluation was completed by 10 of the 14 Coalition members (71% response rate). Overall, Coalition member satisfaction with the experience was high (see Table 2). Ratings were particularly high for quality of facilitation, format of the review process, approach to the reviews, and staff support. Members were slightly less satisfied with different aspects of the materials provided for review (e.g. videos, written materials), although ratings were still generally good. Open comments suggested that the materials need more focus on plain language and should include more graphics where possible. There was a good level of satisfaction with the stipend provided, and a high level of satisfaction with staff support provided (90% strongly satisfied). Open comments reflected that members find the meetings to be efficient and well-organized, and that there is a high degree of transparency.

**Table 2.** Results of coalition member evaluation (n = 10)

	Strongly satisfied/agree	Somewhat satisfied/agree	Neither satisfied/agreed or dissatisfied/disagree	Somewhat dissatisfied/disagree	Strongly dissatisfied/disagree
Overall satisfaction	80%	20%	0%	0%	0%
Review process	80%	20%	0%	0%	0%
Quality of facilitation	90%	10%	0%	0%	0%
Materials provided for review	60%	40%	0%	0%	0%
Level of stipend provided	70%	20%	0%	10%	0%
Staff support provided	90%	10%	0%	0%	0%
Ideas taken seriously	90%	10%	0%	0%	0%
Input respected	90%	10%	0%	0%	0%
There is conflict at the meetings	20%	10%	10%	10%	30%
Conflict is dealt with well	30%	30%	10%	10%	0%
The Coalition is valuable for researchers and likely have impact on studies reviewed	90%	10%	0%	0%	0%
The Coalition has value for the community	70%	20%	0%	10%	0%
The Coalition has increased my interest in research	70%	30%	0%	0%	0%
The Coalition has increased the likelihood that I will recommend research participation to others	90%	10%	0%	0%	0%

We also queried members’ experience with the Coalition process. Ratings were high for comfort in making suggestions, feeling that all ideas were taken seriously, and that one’s input was respected by peers, staff, and researchers. There was more dispersion in members’ ratings of there being conflict and tension at the meetings (30% strongly or somewhat agree; 40% strongly or somewhat disagree); however, 75% of members reported that conflict is dealt with well. Several of the open comments reflected that when it was apparent, tension reflected different opinions and experiences that were treated with respect. Other open comments, reflecting discussion in the Coalition meetings, indicated that some members were frustrated that studies reviewed did not address the needs of all under-represented groups (e.g., Asian and LGBTQ populations). There was also concern raised that the review meetings should be extended to 90 minutes to allow for less rushed discussion among members and with researchers.

We were also interested in members’ views of the value of the Coalition for their communities as well as for the research enterprise. Members strongly agreed that the Coalition is valuable for researchers, that it will likely have impact on the studies reviewed, and that it has value for their community. There was also agreement that participation in the Coalition has increased members’ interest in research and that it has increased the likelihood that they would recommend research participation to others.

## Discussion

The development of the Community Coalition for Equity in Research reflects the desire of our CTSA, together with key community partners, to re-imagine the way in which our researchers engage with community. Our primary goal is to create authentic 2-way engagement and learning opportunities for both researchers and community members, with a primary focus on improving research in ways that will maximize both participation and equitable health outcomes. It is also our goal to provide input that will not only impact on the specific study reviewed but also generalize to an investigators’ entire portfolio as well as to their collaborative activities. Creating such a mechanism for engagement requires infrastructure and time – that of the leadership, our researchers, and our Coalition members. Our early experience, catalyzed by the recruitment challenges faced by the COVID vaccine trials, suggests that this is well worth the investment.

The evaluation of researcher experience indicated that the Coalition adds value as it will improve their research. There were a number of specific areas in which Coalition input was particularly valuable, including identification and engagement with community partners, recruitment strategies, and tailoring opportunities for participation in ways that are inclusive of low resource communities. Feedback suggested that more time for engagement with the Coalition would be helpful. It has been a bit challenging to engage investigators early in their study development process. We have already observed an improvement in this regard, as we are getting the word out more broadly about the Coalition. It has also been challenging to balance supply and demand in terms of maintaining a manageable review volume. Our goal is to have enough studies to keep the Coalition active and to have a study to review each month, but not so many requests that investigators face a substantial time delay.

Overall satisfaction with the Coalition was high among members, as was satisfaction with the review process. Areas identified for improvement included the research materials provided for the review (e.g., consent forms and recruitment flyers that are more accessible to a broad audience), as well as the length of the review meetings. It was interesting that some members noted that there is tension or conflict in the review meetings, but that it is handled effectively. Open comments suggest that this reflects a healthy tension when diverse perspectives are shared and processed, as do the very high ratings of member agreement that they are treated with respect and their ideas are welcome. Our community co-leaders have advised that a lack of tension would indicate more limited engagement or an assumption that different opinions should not be shared. We will continue to evaluate this to ensure that the tension is in fact productive. It is the perception of the Leadership Team that the identity of the Coalition improves at every session, and that there are high levels of engagement. This engagement is reflected in meeting attendance, which averages 12 of 14 members each month.

There are several design features that we believe have facilitated the development of a well-functioning and effective coalition. First, from the very beginning we have adopted an iterative, co-creation approach to the Coalition structure and review process. This has enabled us to quickly build trust and engagement among the group. Second, we have a highly skilled facilitator on our Leadership team, which has enabled the Coalition to address challenging issues in ways that are mutually respectful and engaging. Having a consistent approach to facilitation across sessions has also added value to our work. Third, we held a de-briefing session at the mid-year point to identify issues that needed to be addressed or tweaked and incorporated ideas generated into our subsequent approach. For example, we originally planned to use alternating reviewer and observer roles only as a training technique to ensure adequate feedback from multiple perspectives. However, this approach was reported to be so valuable that it was integrated as a permanent part of the process. Finally, the Coalition’s structure has effectively created a safe space for “productive tension,” in which researchers and Coalition members with the common value of promoting equity in research can come together for facilitated conversations where new, discordant, and potentially uncomfortable perspectives can be explored and discussed. The structure of the meetings seeks to shift the power to prioritize the voices and perspectives of the Coalition members, while also allowing researchers the opportunity to describe the constraints of their research and ask the Coalition-specific questions.

The Coalition has already provided some tangible benefits to our research community. First, its regional representation is resulting in significantly increased awareness about the Boston-centric nature of much of our research enterprise and providing some helpful pressure for the research community to develop ideas to address this. Second, we anticipate that some Coalition members may become directly engaged in research, and we continue to lay the foundation for this to happen. For instance, Coalition members have been invited to join the membership of the Harvard University Area IRB, which will provide the IRB with community members who have basic grounding in study design and who understand and center equity. Third, for studies that are perceived as addressing important community priorities in ways that address equity issues, Coalition members are opening their networks to investigators, facilitating connections and offering ongoing support. Fourth, the Coalition members have encouraged investigators to address barriers that may go beyond their authority or financial capacity, such as going back to funders to request resources needed to address equity considerations. Finally, the Coalition has provided input to investigators on strategies that had not been on their radar. For example, feedback has been provided about the need for more welcoming recruitment approaches and the importance of using local vendors in order to provide ancillary economic benefits to the local community. This feedback led one investigator to hire a local graphic designer from the target community to develop recruitment materials and to create a community-based staff position focused on recruitment. Another investigator who is conducting a study focused on genetic screening in children heard about the importance of involving the child’s health care provider directly in the research discussions, rather than expecting parents to serve as the bridge between the research and their child’s care team. This team also heard recommendations about addressing the impact of genetic findings on parents’ concerns, including the implications for other children in the family.

Our experience with the Coalition thus far is that it has had a positive impact on community member engagement in activities related to research and has brought significant value to our investigators. We developed processes designed to “walk the talk” with shared leadership, respect, compensation for community member time, and co-creation as underlying principles of all of our activities. Coalition members have been actively engaged, and both investigators and Coalition members are beginning to refer others to the Coalition. We are engaged in active conversations with several local IRBs about ways that the Coalition can support their efforts to improve the equity implications of their institution’s research program. We are optimistic that the Coalition will continue to develop and grow into a vibrant entity that will bring value to both investigators and our local communities and will increase the consideration of equity as a foundational principle in all translational research.

## Supplementary material

For supplementary material accompanying this paper visit https://doi.org/10.1017/cts.2022.396. click here to view supplementary material
