# Supplementary material for: Enabling community input to improve equity in and access to translational research: The Community Coalition for Equity in Research
Source: J Clin Transl Sci. 2022 Apr 29;6(1):e60. doi: 10.1017/cts.2022.396 (PMC9161041; doi:10.1017/cts.2022.396)

**Supplemental Materials**

This Glossary is primarily intended for community partners to familiarize with common acronyms and research terms used in community-engaged research and practice. With a focus on health equity and inclusion, alternative research terms are also considered as a way to facilitate better understanding and communication between community partners and researchers. Original definitions were drawn from sources cited and adapted to ensure accessible language. This is a living document which will be regularly updated to reflect new resources and terminology that may be useful for community partners.

ACRONYMS

A list of useful public health terms and organizations.

| ACA | Affordable Care Act |
| --- | --- |
| ACO | Accountable Care Organization |
| ADA | Americans with Disabilities Act (U.S. Department of Labor); also American Dental Association; American Diabetes Association; or American Dietetic Association |
| AHRQ | Agency for Healthcare Research and Quality |
| AMA | American Medical Association |
| AMCHP | Association of Maternal and Child Health Programs |
| AMSA | American Medical Student Association |
| ANA | American Nurses Association |
| AoA | Administration on Aging (HHS) |
| ASTHO | Association of State and Territorial Health Officials |
| CAB | Community Advisory Board |
| CBHI | Children's Behavioral Health Initiative |
| CBO | Community-based Organization |
| CBPR | Community-based Participatory Research |
| CCA | Commonwealth Care Alliance |
| CDC | Centers for Disease Control and Prevention |
| CEnR | Community Engaged Research |
| CHAMPS | Community Health Center and Academic Medical Partnerships |
| CHC | Community Health Center |
| CHIP | Community Health Improvement Plan, or Children’s Health Insurance Program |
| CHNA | Community Health Needs Assessment, or Community Health Network Area |
| CMS | Centers for Medicare & Medicaid Services |
| DCF | Department of Children and Families |
| FDA | Food and Drug Administration |
| FMLA | Family Medical Leave Act (U.S. Department of Labor) |
| FPL | Federal Poverty Level |
| FQHC | Federally-Qualified Health Centers |
| HCR | Health Care Reform |
| HHS | U.S. Department of Health and Human Services |

| HIM | Health Insurance Marketplace |
| --- | --- |
| HIPAA | Health Insurance Portability and Accountability Act |
| HIX | Health Insurance Exchange |
| HRSA | Health Resources and Services Administration |
| IHI | Institute for Healthcare Improvement |
| IHS | Indian Health Service |
| IRB | Institutional Review Board |
| LGBTQIA | Lesbian Gay Bisexual Transgender Queer Intersex Asexual |
| MassCHIP | Massachusetts Community Health Information Profile |
| MCAD | Massachusetts Commission Against Discrimination |
| MDPH | Massachusetts Department of Public Health |
| MOD | Massachusetts Office on Disability |
| MOU | Memorandum of Understanding |
| NACCHO | National Association of City and County Health Officers |
| NACHC | National Association of Community Health Centers |
| NIH | National Institutes of Health |
| ODEO | Massachusetts Office of Disability and Equal Opportunity |
| OHE | Office of Health Equity, Massachusetts Department of Public Health |
| PCORI | Patient-Centered Outcomes Research Institute |
| PI | Principal Investigator |
| SDOH | Social Determinants of Health |
| WIC | Women Infants and Children |

RESEARCH TERMS

A list of commonly used research terminology.

**A**

**Academic-Community partnership:** A partnership between communities and academic institutions where they share power and resources. They work towards change that helps everyone involved. (Duke CTSI)

**Attrition:** When people stop participating in a study. For example, people might drop out of a study because of a change in their health status, or they might lose interest or move away. (PCORI)

**B**

**Beneficence:** Researchers should make the participant's well-being a goal of the research study. The benefits of the research should be bigger than the risks. (RAND)

**Bias (in research):** Bias happens when the differences between groups might affect the results of the study. This makes the study less trustworthy. For example, a study might compare patients at Clinic A taking Medicine Y with patients at Clinic B using Medicine Z. However, most patients at Clinic A are older than 65. Most patients at clinic B are between ages 30 and 45. If Medicine Z looks like it works better, it might be because of the medicine, or it might be because patients who got it were younger and healthier. Bias can also happen when people in a study don't reflect the larger population. (PCORI)

**C**

**Clinical research:** Studies how safe and effective new medicines, treatments, and medical devices are for human use. (Duke CTSI)

**Clinical significance:** The effect of a treatment is enough to make a difference in decisions about treatment. For example, results of a study might suggest different doses of medicine be used. (PCORI)

**Clinical trial:** A study that looks at how safe it is for people to take medicines or treatments and how these medicines/treatments affect the body. (PCORI)

**Clinical trial agreement**: An agreement or contract between researcher and a funder that explains how researchers will do the clinical trial. (Duke CTSI)

**Cluster randomized trial:** A type of study design in which the researchers randomly assign a treatment to a group of people with something in common. For example, all patients who all go to one clinic or all students who all go to one school would get the same treatment. The treatment would not be randomly given to individuals. (PCORI)

**Coercion:** Motivating people to join a study because of a real or imagined threat. For example, students might be afraid of getting a lower grade in class if they don't take part in their teacher's study. Prisoners might think they will be released sooner if they join a study. People should be able to join studies freely. They should not be afraid of negative consequences. (PCORI)

**Cohort:** A group of people in a research study. (PCORI)

**Cohort study**: A type of research design that follows groups of people over time.

**Co-Investigator (CO-I):** A person who works with the study leader on the planning or conduct of a research project. (Duke CTSI)

**Community Advisory Board:** A group that is made of a variety of community members with different experiences. They provide input for researcher on the planning and implementation of studies. (Duke CTSI)

**Community-based Participatory Research:**A type of research where community members and researchers work together on all parts of the research process. Everyone is able to share their experience, expertise, and make decisions together. (Duke CTSI)

**Community capacity building:** Capacity building is helping to improve the work of a community group or its members. Ways to build capacity include providing new information or skills, or providing resources to improve technology. (Nat’l Council on Nonprofits)

**Community engagement:** This is a partnership that involves an active relationship between researchers and members of the community. It promotes an exchange of information, ideas, and resources. Community engagement can include different levels of involvement, decision-making, and control. (Duke CTSI)

**Community engaged research:** An approach to research that involves researchers working with different communities and organizations. These communities may be groups of people who are connected by location, identity, special interest, or other factors. It is a powerful approach that can bring about change in policies, programs, and practices for the community's benefit. (Harvard Catalyst CEP)

**Community health workers:** Health professionals that provide informal counseling and social support. They educate community members on health matters and advocate on their behalf. Community health workers also offer referral and follow-up services to their clients. Research shows that community health workers improve health outcomes many diverse populations. They are a bridge between clients and health services. Community health workers improve access to primary health care and improve quality of care. Their work helps to reduce costs of care and health disparities. (CLAS, Mass.gov)

**Comparative effectiveness research:** This is a type of research that compares two or more treatment options. The goal is to see which works better for certain patients under certain conditions. For example,

a researcher might compare two headache medicines to see which one works better for older women who get very bad headaches. Comparative effectiveness research is often referred to as CER. (PCORI)

**Confidentiality:** Researchers will protect the participant's private information. Confidentiality is similar to privacy. Participants should understand and agree to the ways researchers will handle private information. (RAND)

**Conflict of interest:**A situation where someone would personally benefit from the study results. For example, a researcher who has a patent on a drug would benefit if the drug was found to be effective. (Duke CTSI)

**Confounding:** When the results of the study could be caused by something other than the study conditions. For example, a study might compare two ways to reduce obesity in children and test those two ways in different cities. If one city also started a youth sports program at the same time, the study results in that city might be affected. This would make it difficult to know how well the obesity program worked. (PCORI)

**Consent forms:** Information about the research project and what is being asked of volunteers. It should give all the important information so that someone can decide whether or not they want to participate. (Duke CTSI)

**Cultural broker**: A person who can help mediate between groups of people with different cultural backgrounds. They may help solve problems between the different groups or speak up for a person/group. Cultural brokers can also be people from the medical field who can solve problems between patients and the health system. They use both cultural and health science knowledge to do this. (CLAS, Mass.gov)

**Cultural competence:** Behaviors, attitudes, and policies that allow effective work in cross-cultural situations. The capacity to value diversity, self-assess personal biases, manage differences, build cultural knowledge, and adapt to diversity and culture of the communities served. (CLAS, Mass.gov)

**D**

**Data:** Information that is collected and analyzed to answer a specific question in a research study. Data can come from many different sources such as surveys, interviews, medical records, insurance claims, and more. (PCORI)

**De-identified data:** When nothing is included in data that identifies a specific person. For example, de-identified data would not include the name, date of birth, place of birth, or address of a study participant. (Duke CTSI)

**Dissemination:** Sharing the study results with different groups. The goal is for the study results to inform policy, practice, and individual choices. (PCORI)

**Dissemination research: T**his is a type of research that looks at the best ways to share research findings. (Duke CTSI)

**E**

**Effectiveness:** The study treatments are found to work as planned in real world settings. (CLAS, Mass.gov)

**Efficacy:** The study treatments are found to work as planned in ideal settings. (Duke CTSI)

**Evaluation:** The process of collecting, analyzing, and using data to look at how successful a program is. (CDC)

**Evidence:** systematic data collected to establish facts and reach conclusions (BMJ Best Practice)

**Evidence-based:** A treatment has been shown to work in research studies. (Duke CTSI)

**Exclusion criteria:** Characteristics that do not allow someone to be a part of the study. For example, a study could be looking only at adults. Kids would not be able to participate. (Duke CTSI)

**Experimental design:** A type of research method where the researcher assigns one or more treatments to people. Then they measure how the treatment affects the person's health. (PCORI)

**F**

**False negative:** A test result that incorrectly shows a negative finding. For example, a lab tests shows no infection, but the patient does have strep throat. (PCORI)

**False positive:** A test result that incorrectly shows a positive finding. For example, a mammogram report identifies a cancer, but it turns out to be a cyst. (PCORI)

**Federally-Qualified Health Centers**: Those centers that receive cost-based reimbursement for services delivered to patients who qualify for Medicaid or for Medicare. In order to qualify, a clinic must receive federal funds or meet other criteria.

**Focus group:** A way to collect data. Volunteers are asked to participate in a group discussion. Researchers ask questions about experiences or opinions on a specific topic. Participants answer those questions and talk as a group. Researchers look for common themes and experiences. (PCORI)

**G**

**Generalizability:** Seeing how well the study result can apply to all other people who have the same condition or circumstance. (PCORI)

**H**

**Health disparities:** The differences in health status among various people. Some groups face higher burdens of illness because of their gender, race, ethnicity, education, income, disability, or where they live. (Duke CTSI)

**Health equity:** Everyone has a fair and just chance to reach their full health potential. No one should be at a disadvantage because of their gender, race, ethnicity, education, income, disability, or where they live. (CLAS, Mass.gov)

**Health impact assessment:**A health impact assessment (HIA) uses different data sources and analytic methods and considers input from stakeholders to determine the likely effects of a policy or program on different groups. An HIA gives ideas to handle those effects. (Duke CTSI)

**Health Insurance Portability and Accountability Act of 1996 (HIPAA):** A U.S. law designed to provide privacy standards to protect patients' medical records and other health information provided to health plans, doctors, hospitals and other health care providers. (Duke CTSI)

**Health literacy:** A measure of how well people can access and understand basic health information so that they can make informed decisions about their health. (CLAS, Mass.gov)

**Health outcome:** When a person, group, or population's health status changes because of an intervention. (Duke CTSI

**I**

**Implementation:** The process of putting a decision or plan into effect. (Duke CTSI)

**Implementation science:** **The goal is to speed up the adoption and use of evidence-based practices, programs, and policies in healthcare. Implementation science helps to fill the gap between research and practice. It brings programs that work to communities who need them. (Harvard Catalyst CEP)**

**Implementation strategies:** These are specific ways to adopt, use, and sustain evidence-based practices, programs, and policies. (Harvard Catalyst CEP)

**Incentive:** A promised reward for participating in a study. The incentive may be financial (such as gift cards), or non-financial (such as class credit or an award ceremony). (PCORI)

**Inclusion criteria:**Characteristics that someone must have in order to participate in a study. For example, a study may look at heart problems in females over 40 years old. The inclusion criteria might be that someone has to be a female and over 40 years old. (Duke CTSI)

**Informed consent:** This is a process where someone learns about the goal of the study, what they would have to do in the study, any potential risks, and any benefits to taking part in the study. Researchers also explain how they would protect the participants' privacy. Once the person learns about the study, they can consent to participate, or choose not to. Informed consent is required for all research studies that involve humans. Anyone who is thinking about joining the study can ask the researcher any questions. (PCORI)

**Institutional Review Board (IRB):** An independent group that reviews and approves the plans, protocols, and materials of a research study. This is to make sure that all the rights of the participants are protected. The group is made up of scientists and community members who are not scientists. (PCORI)

**Intervention:** A healthcare prevention, diagnosis, treatment, or delivery activity that is being studied. (PCORI)

**Investigator:** A person who thinks about and carries out a specific research study. (Duke CTSI)

**L**

**Limited English Proficient:** A person who does not speak, read, write, or understand English at the same level as health and social service providers. It may be hard to communicate their health needs. (CLAS, Mass.gov)

**Linguistic competence:** The ability of an organization and its staff to communicate information to a wide variety of people. For example, they should be able to communicate information to people for whom English is not their main language. The organization should also be able to communicate information to those who do not read or write well, and those with visual and hearing difficulties. (CLAS, Mass.gov)

**M**

**Measure:** A specific outcome or result that the research team chooses as a way of answering the research question. Measures are based on data that the research team can collect in consistent ways. For example, a team might want to compare two blood pressure medicines. One measure might be patients’ blood pressure after three months of taking the medicine. Another measure might be how many patients had a heart attack or died after starting the medicine. Another measure might be patients’ feelings about their health after taking the medicine for a year. (PCORI)

**Memorandum of Understanding:** An agreement between two (bilateral) or more (multilateral) parties. (Similar to **Memorandum of Agreement**) (Duke CTSI)

**Method:** A scientific process or plan for how a research team should answer a research question so that the findings are valid, reliable, and credible. A method lays out what kind of data to collect and how to

collect it. Methods also help researchers analyze the data, or understand what the data means and how it answers the question. (PCORI)

**Minimal risk:** The chance of harm from the research is not bigger than the chance of harm from normal daily activities. The chance of harm is also not bigger than the chance of harm from routine medical tests. (Duke CTSI)

**Mixed methods:** A type of study that uses both qualitative and quantitative methods to answer a research question. For example, a research team that wants to understand community weight loss programs might first conduct focus groups (qualitative methods) to get people's ideas about what kinds of weight loss programs they would like to have in their town. In the second part of the study, the research team would assign people to those weight loss programs. The team would then use quantitative methods to see whether people lost weight in each program. (PCORI)

**Monitoring:** Observing and reviewing the progress and quality of something over a given amount of time. (Duke CTSI)

**N**

**Nonexploitation:** Researchers should not take unfair advantage of research participants. (RAND)

**O**

**Observational study:** A type of study in which researchers do not assign the treatment to people. Instead, they look at how the treatment impacts health outcomes in regular health care. (PCORI)

**Outcomes:** Research results that one can measure. For example, a diagnosis, like a broken bone on an x-ray, is an outcome. Another example of an outcome is a change in health like how long a patient lived or if they felt better. (PCORI)

**P**

**Patient-centered care:** Health care that promotes a partnership with patients and their families. The goal is to make sure decisions are made that respect and honor patients' wants and needs. This approach to care also helps to educate and support patients. (CLAS, Mass.gov)

**Patient-centered outcomes research:** A type of comparative effectiveness research. It compares results that matter most to patients and caregivers. Patients and caregivers must be active partners in the research. they make the research more relevant and useful by sharing their lived experiences. (PCORI)

**Personally Identifiable Information (PII):** Information that can be used to identify, contact, or locate a person. (Duke CTSI)

**Population:** A group of people with one or more common characteristic. This could be their health condition, age, race, gender, job, or geographic location. For example, a population in a research study might be men who smoke or women in their 20s. (PCORI)

**Population health:** The health outcomes within a group of people rather than of each person. (New York State)

**Power calculation:** A study needs to have enough people to detect the difference between two treatments. Power calculation is using statistics to determine the right number of people for the study. (PCORI)

**Principal Investigator:** The person who leads and organizes the research study and the team. (PCORI)

**Privacy:** Research participants have the right to control access to their personal information. Participants also have a right to control the collection of samples from their bodies. They can control how other people see, touch, or get their information. (RAND)

**Probability value (p-value):** A statistical concept about the probability that the study would produce a result by chance, even if there were no difference between the two treatments. A p-value of .05 means that there is a 5 percent, or 1-in-20 chance, that a difference in results for Medicine A and Medicine B is just chance. The lower a p-value, the more confident researchers are that there is a real difference between two treatments. (PCORI)

**Protected Health Information (PHI):** The data a medical provider collects to figure out best care for a patient. They may get data includes demographic information, medical history, test and laboratory results, and insurance information. (Duke CTSI)

**Protocol:** A series of steps the research team will follow to do the research study. A protocol explains who will be in the study, and where, when, and how data will be collected. It also explains how the researchers will protect participants' rights. An Institutional Review Board (IRB) reviews protocols. (PCORI)

**Public health:** Public health promotes and protects the health of people and the communities where they live, learn, work and play. (APHA)

**Q**

**Qualitative research:** Research methods that use people's thoughts to answer questions. Some ways of getting this data include interviews and focus groups. for example, focus groups could help a researcher understand how to improve mental health services in schools. Another study could use interviews to find out what matters most to people living with a diabetes. (PCORI)

**Quantitative research:** Research methods that use numbers to measure the relationships between treatments and how they affect health. Examples of quantitative measures include blood pressure readings, survey responses on a rating scale, or the number of days spent in the hospital. Researchers

analyze the data using statistics to figure out the relationship between a treatment and the result. For example, they might test whether patients spent less time in the hospital with Treatment A than with Treatment B. (PCORI)

**R**

**Racial bias:** A previously formed negative opinion or attitude towards a group of people who have common skin color, physical, or cultural characteristics. (CLAS, Mass.gov)

**Randomization:** A process used in experimental study designs. Patients are assigned to treatment groups by chance. (PCORI)

**Recruitment:** The process of inviting people to participate in a research study. The research team identifies potential study participants. They will explain all parts of the study. The research team will also explain any benefits or harms that may occur during or after the study. Then the team will ask participants if they would like to take part in the study. (PCORI)

**Registry-based research:** An organized system that uses observational methods to collect uniform data. This is to evaluate specific outcomes for a population that is defined by a disease, condition, or exposure. It serves one or more scientific, clinical, or policy purposes that was decided ahead of time. (Duke CTSI)

**Reliability:** The degree to which, if another research team did the same study using the same methods, they would get similar results. (PCORI)

**Research integrity:** Researchers should be honest and truthful. They should not make up data, change results, or hide relevant data. They should report all their results and try to minimize bias in the methods. (RAND)

**Retention:** Keeping participants involved at each step of a study. (PCORI)

**S**

**Sample:** A group of people participating in a study. Researchers decide on the characteristics that are important to include. They want to make sure the sample is a good match for the groups that they are trying to study. For example, they may want to get people of different ages, races, and ethnicities. (PCORI)

**Sample size:** The number of participants in a study. The sample size should ideally be large enough for a study to detect differences between two or more treatments. (PCORI)

**Screening:** A test that detects signs of a condition in people who do not have symptoms. For example, a doctor might use a blood test as a screening method for early signs of diabetes. Based on the results of screening, a patient might get more tests to confirm that they have a condition. (PCORI)

**Setting:** Where a study takes place. For example, a study about preventing falls at home might look different than a study about preventing falls in a nursing home. (PCORI)

**Social Determinants of Health:** Where a person is born, lives, learns, works, plays, or worships can affect a wide range of health outcomes and risks. (Duke CTSI)

**Statistics:** The science of analyzing data based on numbers. This helps researchers to look at the relationship between treatments and results in a study. For example, they can figure out whether results are probably caused by a treatment or random chance. (PCORI)

**Statistical significance:** The chance that a study result is caused by the treatment being studied. (PCORI)

**Study design:** A plan or series of steps in order to do research study. This helps the researcher turn their idea into a hypothesis they can test. (Duke CTSI)

**Systematic review:** A type of study that looks at all the evidence that can be used to answer a research question. For example, a research team might do a systematic review of all previous studies about how well a medicine worked. The reviews are called systematic because they follow strict rules about the quality of evidence they include and how the results of different studies can be combined. (PCORI)

**T**

**Timeframe:** The time it takes for a research study to happen. For example, how long patients get a treatment for or how long the research team collects data. (PCORI)

**Translational research:** The process of applying basic biology knowledge and clinical trial results to strategies that address medical needs. The goal is to move basic science discoveries to practice use to improve health outcomes. (Duke CTSI)

**U**

**Underserved populations:** Populations with a lack of access to primary care services. (Duke CTSI)

**V**

**Validity:** How well a study measured what it wanted to measure. There are several types of validity. Internal validity is being able to demonstrate that the results are based on the treatment and not on other factors. External validity is being able to apply the results of the study to other people or other settings. Content validity is making sure to measure all the parts that make up the studied thing. For example, a study about depression would not just ask about mood, but about energy level, self-worth, and disrupted sleep or eating. (PCORI)

**Variables:** Something that can be measured and have different values. It might play a role in study results. For example, many studies collect information about patients' age, race, ethnicity, and sex. Each

is a variable. The research team might compare results for men and women, or people younger than or older than 65. (PCORI)

**W**

**Withdrawal of consent:** A person who is taking part in a research study can decide to stop participating at any point during the study. (Duke CTSI)

HEALTH EQUITY RESEARCH TERMS

A list of alternative research terms that foster equity and inclusion in community-engaged research.

**Belonging** (versus ***Inclusion***): Use language that shows equal partnership in the research process. For example, "we want you to be a part of this study" vs. "you belong in the conversation now and always."

**Different groups** (instead of ***Subgroups***)**:** Groups of study participants who have a common characteristic. For example, a study might focus on how well a blood pressure medicine works for people older than age 65. The research team might also look at how well the medicine worked for specific groups within the study sample. They could look at how well the medicine worked for women older than 65 or people who have diabetes and are older than 65. (PCORI)

**Historically disadvantaged groups/communities** (instead of ***Vulnerable groups***): Groups and communities that are left out or discriminated against because of unequal power relationships in society. (National Collaborating Centre for Determinants of Health)

**Study participant** (instead of ***Human subject***): A person who participates in a research study.

**Under-resourced** (instead of ***Low-income***): Communities or people who have fewer resources compared to the general population. However, in some cases (for example, poverty research), "low-income" may be appropriate to show specific income or poverty levels.

LOCAL RESOURCES

A list of Massachusetts’ organizations that intersect with public health and health equity work.

- [Massachusetts Association for Community Action](https://www.masscap.org/)
- [Massachusetts Association of Health Boards](https://www.mahb.org/)
- [Massachusetts Association of Public Health Nurses](https://www.maphn.org/)
- [Massachusetts Department of Housing and Community Development](https://www.mass.gov/orgs/housing-and-community-development)
- [Massachusetts Department of Public Health](https://www.mass.gov/orgs/department-of-public-health)
- [Massachusetts Environmental Health Association](https://maeha.org/)
- [Massachusetts Health Officers Association](http://www.mhoa.com/)
- [Massachusetts League of Community Health Centers](http://www.massleague.org/)
- [Massachusetts Office of Community Health Workers](https://www.mass.gov/community-health-workers)
- [Massachusetts Office of Rural Health](https://www.mass.gov/state-office-of-rural-health)
- [Massachusetts Public Health Association](https://mapublichealth.org/)
- [New England Rural Health Association](https://nerha.memberclicks.net/)

NATIONAL RESOURCES

A list of federal agencies and major national organizations that intersect with public health and health equity work.

- [Agency for Healthcare Research and Quality (HHS)](https://www.ahrq.gov/)
- [American Public Health Association (APHA)](http://www.apha.org/)
- [Association of Public Health Nurses (APHN)](http://www.phnurse.org/)
- [Association of Schools & Programs of Public Health (ASPPH)](http://www.aspph.org/)
- [Association of State and Territorial Health Officials (ASTHO)](http://www.astho.org/)
- [CDC Office of Science and Public Health Practice](https://www.cdc.gov/cpr/science/index.htm)
- [CDC Public Health Professionals Gateway](https://www.cdc.gov/publichealthgateway/index.html)
- [County Health Rankings and Roadmaps](http://www.countyhealthrankings.org/)
- [Health Impact Partners](https://humanimpact.org/)
- [National Association of City and County Health Officials (NACCHO)](http://www.naccho.org/)
- [National Association of Local Boards of Health (NALBOH)](http://www.nalboh.org/)
- [National Institutes of Health (NIH)](http://www.nih.gov/)
- [National Partnership for Action - The Office of Minority Health](https://www.minorityhealth.hhs.gov/)
- [Public Health Accreditation Board](http://www.phaboard.org/)
- [Public Health Foundation](http://www.phf.org/)
- [Robert Wood Johnson Foundation Culture of Health](https://www.rwjf.org/en/how-we-work/building-a-culture-of-health.html)
- [Society for Public Health Education (SOPHE)](http://www.sophe.org/)
- [U.S. Centers for Disease Control and Prevention (CDC)](http://www.cdc.gov/)
- [U.S. Centers for Medicare & Medicaid Services (Medicaid)](https://www.medicaid.gov/)
- [U.S. Centers for Medicare & Medicaid Services (Medicare)](https://www.medicare.gov/)
- [U.S. Department of Health and Human Services (USDHHS)](http://www.dhhs.gov/)

Supplemental Materials: Health Equity Rubric


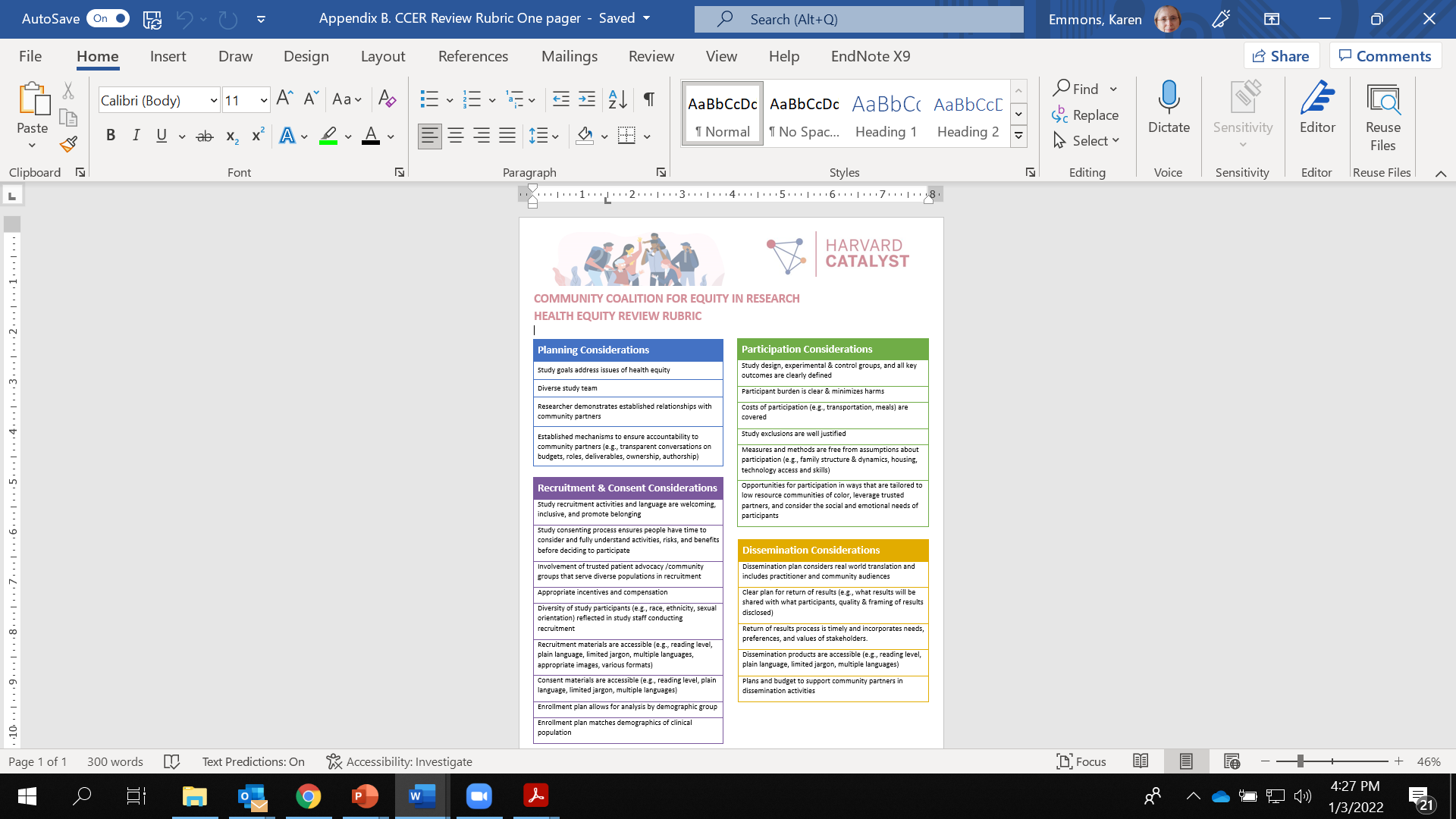

Supplement: Supplementary file 1 [file S205986612200396Xsup001.docx]
